# Sacroiliitis: A Review on Anatomy, Diagnosis, and Treatment

**DOI:** 10.1155/2022/3283296

**Published:** 2022-12-28

**Authors:** Anderson Lee, Monik Gupta, Kiran Boyinepally, Phillip J. Stokey, Nabil A. Ebraheim

**Affiliations:** Department of Orthopaedic Surgery, University of Toledo College of Medicine and Life Sciences, 3000 Arlington Ave, Toledo, OH 43614, USA

## Abstract

**Introduction:**

Sacroiliitis is an inflammation of one or both of the sacroiliac (SI) joints, most often resulting pain in the lower back that can extend down the legs. Pain arising from the SI joint can be difficult to diagnose and treat due to the intricate surrounding ligamentous structure, nerve innervation, and its role in transferring weight from the upper body to the lower limbs. SI joint dysfunction accounts for up to 25% of cases of lower back pain and has a debilitating effect on patient functionality. This review aims to provide comprehensive coverage of all aspects of SI joint pain, with a specific focus on differential diagnosis and treatment.

**Methods:**

Current literature on SI joint pain and inflammation, other etiologies of lower back pain, and new treatment options were compiled using the databases PubMed and Cochrane and used to write this comprehensive review. There were no restrictions when conducting the literature search with regard to publication date, study language, or study type.

**Results:**

The diagnosis protocol of SI joint pain arising from sacroiliitis usually begins with the presentation of lower back pain and confirmatory diagnostic testing through fluoroscopy joint block. Reduction in pain following the anesthetic is considered the golden standard for diagnosis. The treatment begins with the conservative approach of physical therapy and analgesics for symptom relief. However, refractory cases often require interventional methods such as corticosteroid injections, prolotherapy, radiofrequency ablation, and even SI joint fusion surgery.

**Conclusion:**

SI joint pain is a complex problem that can present with varying patterns of pain due to uncertainty regarding its innervation and its prominent surrounding structure. It is therefore especially important to obtain a thorough history and physical on top of diagnostic tests such as a diagnostic block to properly identify the source of pain. Conservative treatment options with physical therapy and analgesics should be attempted first before interventional strategies such as ablation, injections, and prolotherapy can be considered. SI joint fusion surgery is a solution to cases in which previous methods do not provide significant relief.

## 1. Introduction

The sacroiliac (SI) joint is a key component of the body's ability to transfer load between the lumbar spine and the lower extremities. It lies between the sacrum and the ilium of the hip bone bilaterally. The SI joint is a source of lower back pain that may comprise up to 25% of all lower back pain cases [1]. It is only capable of around 1.5 degrees of axial rotation and an average of <2 mm of translation, making it severely limited in its range of motion. Sacroiliac joint pain and inflammation, also known as sacroiliitis, can be caused by sacroiliac joint dysfunction (SIJD). Triggers of joint pain include trauma, pregnancy, stress, lumbar fusion surgery, and bone grafts near the sacroiliac joint [2]. SI joint pain is difficult to diagnose and present similarly to many other causes of back pain. Due to its similar presentation to other varying causes of lower back pain, it is vital for clinicians to identify important hallmarks of pain arising from the SI joint to properly diagnose and treat this condition. This article aims to provide a comprehensive and current review of the anatomy, epidemiology, diagnosis, and treatment pertinent to sacroiliac joint pain stemming from sacroiliitis.

## 2. Anatomy

### 2.1. Bone/Function

The main function of the SI joint is to connect the iliac crests of the hip bones to the sacrum, providing a stable yet flexible support to the upper body while also distributing load from the lower extremities throughout the rest of the body. It is considered the largest axial joint in the body, with an average surface area of 17.5 cm^2^ [1]. The SI joint is a synovial diarthrosis-amphiarthrosis joint and each joint on either side of the sacrum is surrounded by a fibrous capsule [1, 2]. Two different types of cartilage constitute the articulation of each joint. The sacral side is composed of a thicker, hyaline cartilage while the iliac side is made up of fibrocartilage. At birth, the joint is similar to the smooth and flat orientation of the zygapophyseal joints [3]. As the child begins movement, the articular surfaces develop a curvature to allow for greater load bearing and stability. A fully developed adult SI joint has a C-shaped, auricular shape with multiple depressions and ridges. The base of the sacrum is wider both superiorly and anteriorly. These grooves and ridges enhance the stability of the SI joints and protect the joint from various forces both vertically and horizontally [1, 3]. Studies have shown an average translation displacement of the joint of 1.3 mm in females and 1 mm in males. Rotational movement of the joint rarely exceeds 4 degrees [3].

### 2.2. Ligaments and Muscles

Various strong ligamentous connections surround and stabilize the SI joint. The interosseous sacroiliac ligament, one of the strongest ligaments in the body, connects the outer surface of the sacrum to the inner surface of the ilium to form the main connection between the sacrum and the ilium. It prevents forward, downward, and excessive backward movement of the joint [1]. The anterior sacroiliac ligament is a weak and thin ligament that runs over the front of the SI joint and blends with the joint capsule. Its weaker structure increases the susceptibility to pain. A stronger posterior sacroiliac ligament is composed of two parts: the long and short posterior sacroiliac ligaments that connect the posterior superior iliac spine and iliac crest to the sacrum [2]. The posterior sacroiliac ligament prevents counternutation, or posterior-superior movement of the sacrum when the coccyx moves anteriorly to the ilium [1]. Other accessory ligaments of the SI joint that further enhance the overall stability of the joint include the sacrotuberous, sacrospinous, and iliolumbar ligaments. These ligaments of the joint form the greater sciatic foramen where the sciatic nerve runs through and the lesser sciatic foramen. Damage to these ligaments can cause sciatica, a pain that runs down the leg along the path of the sciatic nerve [1, 2].

The muscles that surround the SI joint are not designed to move the joint or produce active movements, but instead, they maintain stability and functionality. There are over 40 muscles that surround the joint including muscles of the back, hip, core, buttocks, and thighs. A sedentary lifestyle can lead to muscle tightness and increased tension around the joint. Thus, adequate exercise and stretching is important to allow the SI joint to function without pain.

### 2.3. Innervation

Innervation of the SI joint is a frequently debated subject, and it is hypothesized that the pattern of innervation varies between individuals. Research suggests that the joint receives innervation from branches of the ventral rami of L4-L5 and dorsal rami of L5-S2, as well as the obturator nerve, the superior gluteal nerve, and the lumbosacral trunk [1]. However, the specific areas of innervation of these nerves remain up for debate, which may account for the varying patterns in referred pain of the SI joint [3].

### 2.4. Epidemiology/Etiology

#### 2.4.1. Epidemiology

Pain arising from the SI joint accounts for up to 25% of cases of lower back pain. The prevalence is higher in lumbosacral fusion surgery patients at 32–37% [4–6]. Many cases arise in younger patient populations following sports injuries or trauma and in pregnant or older patients due to joint degeneration. Women have more flexibility in their SI joint and are therefore more susceptible to SI joint dysfunction due to increased stress, movement, and load on the surrounding structures [5, 6].

#### 2.4.2. Etiology

Causes of SI joint pain can range from traumatic causes such as abrupt rotation, motor vehicle collisions, falls, and pregnancy to atraumatic causes such as prior lumbosacral spinal fusion surgery, ankylosing spondylitis, arthritis, scoliosis, and infection. The characteristics of the SI joint that make it stable and severely limited in its motion also make it vulnerable to numerous causes of stress [7]. Lumbar spine surgery is a risk factor for SI joint pain due to weakening of the surrounding ligamentous structure or damage to the joint cavity due to grafts taken from the iliac bone. Damage to the joint capsule, ligaments, muscles, or surrounding nerve roots can therefore cause various patterns of pain that originate from the SI joint [4, 6].

### 2.5. Clinical Presentation

SI joint pain is often difficult to diagnose as symptoms of the condition may mimic a myriad of other conditions such as facet joint arthritis and piriformis syndrome [4]. Patients often present with a deep-rooted pain that begins in the posterior thigh and extends to the knee or even the entire lower extremity. Pain will usually present non-midline and to the side, below the L5 dermatomal level, which would be below the iliac crest. Patients will point to the posterior superior iliac spine or just below and medial to it when asked where it hurts [7]. This pain may occur when patients are sitting down, walking for long periods of time, climbing stairs, or laying on the affected side [4, 8]. To correctly diagnose a patient with SI joint pain, a thorough investigation of clinical symptoms and medical history must occur. Relevant questions asked by physicians should include those about dietary habits, sleeping patterns, and exercise routines. In addition, examiners must explore any history of trauma, infection, or inflammatory disease such as rheumatoid arthritis and ankylosing spondylitis. It is important to acknowledge that SI joint pain often occurs due to an inciting event and does not emerge gradually.

Utilizing various provocation tests can elicit SI joint pain and these include the following: gait patterns, thigh thrust test, sacral thrust test, distraction test, FABER, palpation tests, compression test, Gaenslen's test, and the Fortin Finger test. These tests are discussed, but it is vital for examiners to recall that there is no golden standard physical exam. It is a combination of provocation tests that creates strong evidence of SI joint dysfunction.

### 2.6. Provocation Tests

#### 2.6.1. Gait Pattern, Leg-Length Inequality, and Lower Lumbar Examination

Patients with SI joint dysfunction often present with an asymmetrical gait due to a reduced activation of the ipsilateral gluteus maximus and contralateral latissimus dorsi [9]. These muscles work together to provide stability and symmetry during walking. Therefore, clinicians should ask patients to walk around the observation room to analyze the gait pattern. It is also important to look for obvious deformities and trauma in the lower lumbar region as these findings may result in SI joint pain. Furthermore, the presence of a leg-length inequality may also cause SI joint pain and is important to investigate [10]. Imaging will play a role in identifying any abnormalities that may not be seen on the physical exam.

#### 2.6.2. Thigh Thrust Test

The thigh thrust test, also known as the posterior shear test, requires patients to lay in a supine position with the clinician flexing the affected hip joint to 90°. Standing on the same side of the flexed joint, the examiner will apply an anteroposterior shear force through the femur axis [11]. If pain is elicited, the test is positive. Laslett et al. determined the specificity of this test to be 69% and the sensitivity to be 88% [12]. In addition, Laslett et al. found the positive predictive value (PPV) to be 58% and the negative predictive value (NPV) to be 92% [12, 13]. The positive predictive value indicates how frequently those with a positive test will actually have the condition, whereas the negative predictive value indicates how often those with a negative test will not have the condition.

#### 2.6.3. Sacral Thrust Test

In the sacral thrust test, the patient will lay in a prone position, and the clinician will place one hand over the other above the sacrum. They will then apply an anterior shear force to the bilateral joints. A positive result is seen if the pain is elicited. Laslett et al. found the specificity of the test to be 75% and the sensitivity to be 63% [12]. Furthermore, the PPV and NPV were found to be 56% and 80%, respectively [12, 13].

#### 2.6.4. Distraction Test

The distraction test will require patients to lay in a supine position, and the clinician will apply a simultaneous vertical and posterior shear force to both anterior superior iliac spines (ASIS). If pain is reproduced, then the test is positive. Laslett et al. found the specificity of the test to be 81% and the sensitivity to be 60% [12]. The PPV was determined to be 60%, and the NPV was found to be 81% [12, 13].

#### 2.6.5. FABER Test

The FABER test is short for flexion, abduction, and external rotation. The three movements are combined simultaneously to elicit symptoms from patients. In this provocation test, the patient lies in a supine position and the affected hip is flexed and abducted, and the lateral ankle of the affected leg is placed on the contralateral thigh, slightly superior to the knee. After this position is achieved, the clinician will stabilize the contralateral ASIS and apply an external rotation, abduction, and posterior force to the ipsilateral knee until the maximum motion is achieved. If pain is elicited or if there is a limit on the motion, then this is considered a positive test. Schneider et al. found the test to have a specificity of 56% and a sensitivity of 50% [14]. Another study determined the specificity and sensitivity to be 66.7% and 71.8%, respectively, and the positive and negative predictive values to be 90.3% and 35.5% [11]. Though these results differ, it is agreed within the literature that the FABER test has the highest specificity and PPV.

#### 2.6.6. Palpation Tests

Palpation tests are by far the most straightforward of the provocation tests because they only require clinicians to apply deep thumb pressure on the SI joints bilaterally. A positive result is seen when pain is elicited. Because the SI joint is below several layers of tissues, the reliability of palpation has been questioned recently. It is argued that eliciting symptoms on palpation is not specific to SI joint dysfunction only and may lead to misdiagnosis [15].

#### 2.6.7. Compression Test

In the sacroiliac compression test, the patient will lay in the lateral decubitus position with the affected side facing upwards. The examiner will then apply a downward pressure towards the floor at the ipsilateral superior aspect of the iliac crest and ASIS. If pain or a sense of intense pressure is felt by the patient at the sacrum, the test is determined to be positive. Laslett et al. found the specificity to be 69% and the sensitivity to be 60% [12].

#### 2.6.8. Gaenslen's Test

In Gaenslen's test, the patient will lay in a supine position, and the affected leg will lay over the side of the table. The unaffected knee will be flexed to the patient's chest and the patient will hold this leg with his or her arms. The clinician will then apply pressure to the flexed knee and counterpressure to the hanging leg. These countering pressures create increased torque at the pelvis. This test is conducted for both sides. The test is positive if pain or the patient's symptoms are elicited during the maneuver [11]. The specificity of this test was determined to be 71% and the sensitivity was found to be 53% by Laslett et al. [12].

#### 2.6.9. Fortin Finger Test

The Fortin Finger test is a simple examination where the patient uses pinpoints where the pain is localized to using one finger. If the pain occurs above the L5 region, SI joint dysfunction is suspected. If the pain occurs below L5, then a lumbar spine pathology is suspected. Since this test is simple and generalized in terms of regions, its reliability has been debated because many pathologies may lead to referred pain in this region [7].

As seen previously, there are numerous tests available to the clinician, and it is important for clinicians to understand how to apply them and also how to form a diagnosis from them. Wu et al. found that patients with SI joint dysfunction were up to twenty times more likely to have a positive result in 3 or more provocation tests [16]. An additional study found that a combination of FABER and thigh thrust test yielded the most accurate results followed by a combination of FABER and Gaenslen test [11]. Interestingly, research has indicated that more than 3 positive provocation tests tend to decrease the positive and negative predictive values [13].

### 2.7. Evaluation and Diagnostics

A SI joint block is the main form of confirmatory diagnostic tests that can truly establish a diagnosis of pain that originates from the SI joint. The fluoroscopy-guided block is the most important test that can be performed for both diagnosing and treating SI joint pain. 1-2 mL of local anesthetic is injected posteriorly, and a reduction in over 75% of pain after the first diagnostic block is considered a positive test [6]. Pain reduction in the 50–75% range would be highly suspicious of some form of SI joint pathology as a contributor to pain. Evaluations with magnetic resonance imaging, computed tomography, or other forms of imaging are not able to reliably identify the cause of pain arising from the SI joint, but rather play important roles in ruling out other sources of pain [6].

### 2.8. Treatment/Management

SI joint pain is treated conservatively before further invasive options such as surgery are considered. Analgesics and NSAIDs may be used for symptomatic pain management. Physical therapy has been demonstrated to yield intermediate and long-term benefits by focusing on spinal stabilization and stretching exercises of the iliopsoas and piriformis muscles. Stretches involving the transversus abdominis muscle may also reduce pain [6]. Chiropractic manipulation and physical therapy are especially beneficial to those with altered gait mechanics and spinal misalignment. Pelvic belts can stabilize the SI joints and reduce sagittal rotation or excess ligament strain, especially in pregnant women with weakened SI joints [4]. Patients with limb-length discrepancy may benefit from shoe inserts to help equally distribute load management of the SI joints. Conservative, noninterventional management should be attempted, and symptoms should be demonstrated to be refractory before injections are administered [5, 6].

Interventional management options for SI joint pain are as follows.

(1) Prolotherapy
  - SI joint pain may result from extraarticular damage to the joint capsule and surrounding ligaments. Injections into areas of the SI joint where strength and repair may be compromised with dextrose and platelet-rich plasma have been shown to reduce pain in patients. Some trials have shown a greater benefit of prolotherapy treatment over steroid injections, though additional research would be needed to establish consistent improvements [4, 6].
(2) Extraarticular/intraarticular injections
  - Corticosteroid injections provide anti-inflammatory properties that aid in relieving clinical symptoms of patients with arthritis, inflammatory conditions, or other musculoskeletal-related causes of SI joint pain. Extraarticular or intraarticular injections can be carried out and should be carried out under fluoroscopy or ultrasound guidance as blind injections only successfully reach the joint spaces 22% of the time [6]. Fluoroscopy and CT-guided injections are the most precise, but ultrasound is more accessible in clinical practice. Most studies show significant pain relief because of injections that can last up to a year [6].
(3) Radiofrequency denervation
  - Radio waves are used to generate an electric current in order to heat and ablate nerve fibers and ultimately reduce pain sensation [6]. The first RF technique reported by Ferrante et al. used in the treatment of sacroiliitis arising from SIJD involved the application of two electrode tips (bipolar type) and only 36% of patients had sustained relief for 6 months [17]. The current conventional method involves unipolar RF lesioning of the dorsal rami lateral branch nerves innervating the SI joint [18]. This method showed improved results with 60% of subjects showing sustained pain relief for 6 months in the associated study [18]. Despite these effects, RF denervation is still limited by its inability to lesion the anterior neural structures of the sacroiliac joint and the potential for nerve regeneration over a period of several months [19]. Other modified versions of this method that are being analyzed include cooled RF ablation, simplicity III RF ablation, and quadrupolar RF ablation [6]. More research comparing these modifications in terms of safety and efficacy is still needed.
(4) Pulsed radiofrequency
  - Although classified under the general category of radiofrequency intervention, pulsed radiofrequency (PRF) does not destroy or damage the nerve tissue, unlike standard RF ablation. This is attributed to the application of RF current in short high-voltage bursts that allow for heat dissipation during the “silent” phases, preventing the start of thermal lesions [20]. Various theories have been postulated regarding the mechanism by which PRF leads to pain relief. A prevalent theory describes the modulation of a c-Fos pathway by alternating electric fields generated by PRF [20]. Another popular theory involves the alteration of the transcription factor ATF3, which ultimately impacts cellular stress in C and A*δ* pain fibers [21]. Although more study is needed to elucidate the mechanisms behind this technique, the short-term analgesic effect is significant. A retrospective study published in 2021 comparing 3 types of radiofrequency techniques (conventional, cooled, and pulsed) for SI joint pain showed the highest rate (100%) of pain relief over a six-month period [21]. It should be noted that other studies have shown varied rates of effectiveness between these methods, and this likely indicates a need for a more comprehensive study with a larger group of patients. Despite these inconsistencies, PRF and RF ablation remain an effective short-term option for significant pain relief in patients with sacroiliac joint pain.
(5) Surgical intervention
  - Sacroiliac arthrodesis (SI joint fusion) has been the established surgical method for recalcitrant cases of pain arising from SIJD. Physiologically, the pain relief is attributed to the removal of the joint space and subsequent limitation of movement [19]. The two primary approaches of SI joint fusion are open arthrodesis and the minimally invasive percutaneous sacroiliac arthrodesis [19]. In the open approach, the joint is accessed anteriorly or posteriorly, cartilage is removed, a bone graft is placed, and plate-screw constructs are added for stability [19]. The minimally invasive approach follows a similar sequence, but the notable differences are in the smaller incision size and the use of radiographic imaging for joint visualization [19]. There has been a trend towards percutaneous arthrodesis in recent years because patients tend to progress towards full weight-bearing ability sooner than the open approach [19]. However, there can be contraindications to the minimally invasive approach and open arthrodesis may be a better option. For instance, patients with a dysmorphic sacrum have a higher risk of neurologic injury and impairment with the percutaneous approach, likely due to the added complexity of the radiographic imaging [19]. Both methods should be considered if conservative options are short-lived or inadequate for pain relief.

### 2.9. Differential Diagnoses

The region of lower back pain caused by SI joint syndrome overlaps with areas associated with a plethora of other conditions. This often leads to difficulty in diagnosing pain arising from the SI joints. Thus, there are several notable differential diagnoses that must be considered to determine whether the sacroiliac joint is the primary source of pain generation. These have been listed in Table 1. Most of these conditions can be categorized by location: either spine or hip. However, general inflammatory arthropathies can present with lower back pain symptoms as well [7].

Systematically excluding the wide range of similarly presenting conditions based on history, physical, imaging, and other tests is imperative for SI joint pain diagnosis. However, there is the possibility that several of the conditions listed in Table 1 can coexist with or influence SI joint pain. For instance, SI joint pain can occur along with piriformis syndrome and cause radicular pain because of the close anatomic proximity of the SI joint and piriformis muscle to the sciatic nerve [22].

## 3. Conclusion

SI joint pain arising from sacroiliitis is a complex problem that can present with varying patterns of pain due to uncertainty regarding its innervation and its prominent surrounding structure. The joint plays an important role in load bearing and is extremely limited in its range of motion, making it susceptible to trauma. It is therefore especially important to obtain a thorough history and physical on top of diagnostic tests such as a diagnostic block to properly identify the source of pain. Many other pathological causes of pain in the lumbar and lower extremity region may present similarly and it is critical to use an image-guided diagnostic block, as it is the golden standard for diagnosing and differentiating sacroiliitis from other sources of pain. Conservative treatment options with physical therapy and analgesics should be attempted first before interventional strategies such as ablation, injections, and prolotherapy can be considered. SI joint fusion surgery is a solution to cases in which previous methods do not provide significant relief. Overall, sacroiliitis is a complex disease that may severely impact a patient lifestyle and functionality, highlighting the importance of a comprehensive physical and diagnosis to properly identify and treat the source of pain.

## Figures and Tables

**Table 1:** Key differential diagnoses presenting similarly with SI joint pain including etiology and clinical presentation.

Differential diagnosis	Brief etiology	Key clinical presentations
*Hip*		
Piriformis syndrome	Compression of the sciatic nerve by piriformis muscle or hypertrophic, tender piriformis leads to neuropathic or myofascial pain, respectively	Ipsilateral radiation of buttock pain to the posterior aspect of one or both legs, exacerbated pain with prolonged sitting
Trochanteric bursitis	Repetitive friction due to overuse or trauma between the IT band and the greater trochanter of the femur can result in inflammation of the trochanteric bursa, located on the lateral aspect of the hip	Lateral hip pain, sleeping on the affected side, and prolonged sitting can exacerbate the pain
Hip fracture	Fracture is often secondary to trauma or osteoporosis	Very limited lumbar range of motion and high pain level, history, and imaging are useful in ruling out a fracture
Hip arthritis	The hip joint is one of the body's largest weight-bearing joints, often impacted by osteoarthritis (OA). OA involves degeneration of the joints with age and affects articular cartilage and/or surrounding tissues of the joint	Reduced mobility and pain around the hip joint and groin are especially common, improved symptoms with movement and activity
Femoral acetabular impingement (hip impingement)	The femoral head of the hip pinches up against the acetabulum, leading to damage of the labrum, pain, and potentially arthritis in the long term	Discomfort while sitting or during prolonged activities

*Spine*		
Lumbosacral disk herniation	Involves the disruption and displacement of the annular tissue of the vertebral body in relation to the nucleus pulposus and involves four main classifications based on displacement: protrusion, extrusion, sequestration, and migration. Various inciting events such as trauma and degenerative genetic factors can lead to disc herniation	Dermatomal radicular pain dependent upon the level of herniation and sensory abnormalities such as tingling and numbness in legs and feet, and leg weakness based on impact in one or more lumbosacral nerve roots
Lumbosacral facet syndrome	Facet joint degeneration occurs secondary to trauma or with age and repetitive overuse. This can lead to microstructural instability and synovial facet cysts that can compress the surrounding spinal nerve roots	Pain from backward extension, radicular pain, and paraspinal muscle tenderness
Spondylolisthesis	This condition directly refers to the slipping of one vertebra in relation to an adjacent vertebra and can be the result of trauma, degeneration, and pathologic causes (i.e., tumor). It can lead to spondylolysis, the fracture of the pars interarticularis	Lower-limb pain is the chief symptom in adults, and radicular and back pain in children

*Other*		
Spondyloarthropathies	They are a group of diseases with a link to the HLA-B27 allele and several phenotypic similarities. Some examples are ankylosing spondylitis, psoriatic arthritis, and reactive arthritis	Inflammatory back pain, extra-articular features such as uveitis, psoriasis, and inflammatory bowel disease, and positive findings on laboratory tests help with diagnosis
Myofascial pain	Trauma or repetitive motion can perturb myofascial trigger points located in the fascia, tendons, or muscles	Paraspinal muscle weakness and radiation of pain to buttocks and thighs
Bone tumor	Osteosarcoma and osteoid osteoma are among several tumors in this category. A variety of molecular mechanisms tied to cancer impact the osteoblasts and osteoclasts, resulting in skeletal instability or destruction	Focal bone pain and pathologic features are often present in imaging

The conditions listed are key differential diagnoses that can present with similar patterns of pain to that arising from the SI joint. These are classified by the location of origin: hip, spine, or other for those that do not have a specific or consistent location. The information compiled to assemble the table was from various sources specific to each of the differential diagnoses [5, 22–30].

## Data Availability

The data used to support the findings of this study are included within the article.

## Abbreviations

SI: Sacroiliac
PPV: Positive predictive value
NPV: Negative predictive value
ASIS: Anterior superior iliac spine
PRF: Pulsed radiofrequency
SIJD: Sacroiliac joint dysfunction.
